# Use of Edoxaban for the Treatment of Heparin-Induced Thrombocytopenia

**DOI:** 10.1155/2020/2367095

**Published:** 2020-09-07

**Authors:** Ryo Kanamoto, Shinichi Hiromatsu, Tomoyuki Anegawa, Kanako Sakurai, Shohei Yoshida, Yusuke Shintani, Hiroyuki Otsuka, Satoru Tobinaga, Hiroyuki Tanaka

**Affiliations:** Department of Surgery, Kurume University School of Medicine, Fukuoka, Japan

## Abstract

Heparin-induced thrombocytopenia (HIT) is a life-threatening adverse drug reaction of heparin therapy, which increases a patient's risk of developing venous and/or arterial thromboembolism. HIT should be treated through discontinuation of heparin and administration of nonheparin anticoagulants such as argatroban. For long-term anticoagulation, parenteral nonheparin anticoagulants are generally converted to oral treatment with a vitamin K antagonist such as warfarin. Although administration of warfarin is recommended to overlap with a nonheparin anticoagulant for a minimum of 5 days, overlapping with argatroban and warfarin presents high risks of bleeding. We describe a case of HIT treated with edoxaban. A 78-year-old man underwent surgery for esophageal cancer and was administered heparin perioperatively. After surgery, he was diagnosed with HIT and venous thromboembolism. We immediately stopped heparin and initiated parenteral argatroban. The patient was subsequently started on edoxaban without any overlap between the two drugs. The treatment was successful. The treatment of edoxaban following argatroban for HIT could reduce bleeding complications and shorten the length of hospital stay. To the best of our knowledge, this is the first report of the use of edoxaban for HIT treatment.

## 1. Introduction

Heparin-induced thrombocytopenia (HIT) is a life-threatening and limb-threatening adverse drug reaction that can lead to venous thromboembolism (VTE), which includes pulmonary embolism (PE) and deep vein thrombosis (DVT). HIT may also lead to arterial thromboembolism, which includes limb ischemia, acute myocardial infarction, and stroke. HIT results from the antibodies against complexes of platelet factor 4 (PF4) and heparin following exposure to unfractionated or low-molecular-weight heparin.

In HIT patients, heparin should be discontinued immediately and replaced with nonheparin anticoagulants such as argatroban, danaparoid, bivalirudin, or fondaparinux. Parenteral anticoagulation treatment is generally converted to oral treatment with a vitamin K antagonist (VKA), such as warfarin, for long-term anticoagulation [1, 2]. Recently, several studies have reported the use of direct oral anticoagulants (DOACs), such as the direct factor Xa inhibitors, rivaroxaban, apixaban, and edoxaban, or the direct thrombin inhibitor, dabigatran, instead of warfarin, as a new treatment option for HIT patients. However, to date, HIT treatment using edoxaban has not been reported [2]. Here, we report the case of an HIT patient with VTE who was successfully treated with edoxaban. To the best of our knowledge, this is the first report that describes the use of edoxaban for HIT treatment.

## 2. Case Report

**Figure 1:**
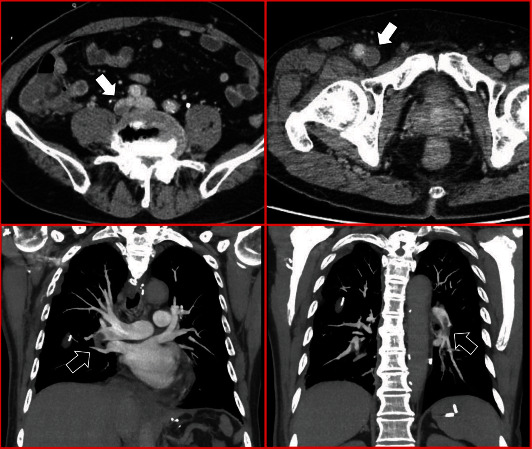
Contrast computed tomography imaging. White arrows indicate the region from the right iliac to the common femoral vein that was occluded by deep vein thrombosis, whereas black arrows indicate the bilateral pulmonary artery thromboembolism.

**Figure 2:**
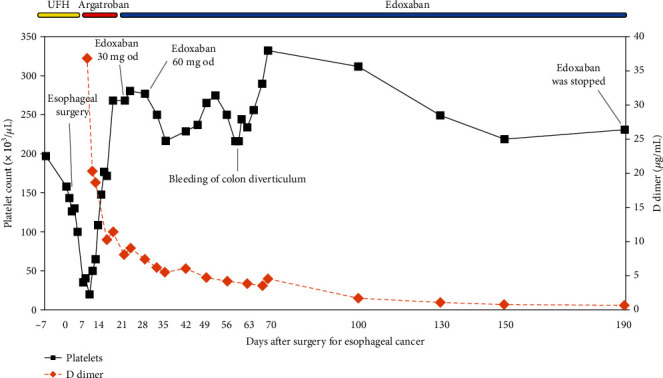
Platelet count and D-dimer value during the administration of anticoagulants in a patient with heparin-induced thrombocytopenia after surgery for esophageal cancer. UFH: unfractionated heparin.

A 78-year-old man was admitted to our hospital for the surgical treatment of esophageal cancer. He had been administered oral clopidogrel for a previous cerebral infarction. His medical history did not show previous venous thromboembolic events. Oral clopidogrel treatment was switched to continuous intravenous unfractionated heparin (UFH) administration (10000 IU once daily) during the preoperative period. Four days after the surgical treatment of esophageal cancer, the administration of UFH was resumed. The patient's platelet count was 197 × 10^3^/*μ*L prior to surgery and 158 × 10^3^/*μ*L immediately after surgery. However, his platelet count decreased to approximately 35 × 10^3^/*μ*L on postoperative day seven. We suspected HIT and immediately stopped treatment with UFH and initiated treatment with intravenous argatroban. Argatroban was initially administered at an infusion rate of 0.35 *μ*g/kg/min because of the risk of bleeding after major surgery. The infusion rate of argatroban was increased to 0.7 *μ*g/kg/min upon monitoring activated partial thromboplastin time (aPTT) (target aPTT is 1.5–2.0× the patient's baseline value; the reagent we used in this case is Coagpia APTT-N, SEKISUI MEDICAL CO., LTD., Tokyo, Japan). Four days after UFH withdrawal, the patient complained of right leg swelling. Contrast computed tomography (CT) revealed bilateral PE and proximal DVT in the right leg (Figure 1). His plasma D-dimer value was 20.3 *μ*g/mL. He underwent placement of a retrievable inferior vena cava (IVC) filter (Günther Tulip Vena Cava Filter, Cook Medical, Bloomington, USA) because of the occurrence of a new PE event during anticoagulant therapy. The 4T's score was 6, and levels of anti-PF4/heparin antibodies were >5.0 U/mL according to the results of the HemosIL® HIT-Ab_(PF4-H)_ latex immunoturbidimetric assay (Instrumentation Laboratory, Bedford, MA). Eleven days after UFH withdrawal and initiation of argatroban treatment, the platelet count gradually increased to 268 × 10^3^/*μ*L and the D-dimer value decreased to 11.4 *μ*g/mL. Because the IVC filter did not capture the thrombus, we removed it 1 week after its placement. Twenty-one days after UFH withdrawal, the platelet count was 268 × 10^3^/*μ*L and the D-dimer value was 8.1 *μ*g/mL. We then stopped the treatment with argatroban and gave the patient edoxaban (30 mg once a day) without any overlap in treatment with the two drugs. As the patient experienced no bleeding complications after 5 days of edoxaban (30 mg) administration, we increased the dose of edoxaban to 60 mg/day. Although the patient suffered from bleeding of the colon diverticulum 38 days after the initial treatment with edoxaban, we performed endoscopic hemostasis and the patient recovered. Later, he did not experience further bleeding complications during edoxaban treatment. We continued his anticoagulant therapy for 6 months to treat cancer-associated thrombosis. Six months after UFH withdrawal, we discontinued treatment with edoxaban because the D-dimer value normalized, PE and DVT disappeared according to the follow-up contrast CT, and there was no evidence of cancer recurrence in any follow-up examinations (Figure 2). At a follow-up appointment 3 years after stopping edoxaban treatment, the patient had experienced neither recurrent thromboembolism nor other complications.

## 3. Discussion

For the treatment of HIT, heparin treatment should immediately be changed to parenteral treatment with a nonheparin anticoagulant. Argatroban is the only parenteral nonheparin anticoagulant that has been approved for the treatment of HIT in Japan. The 9^th^ guideline of the American College of Chest Physicians (ACCP) recommends that argatroban be administered initially at an infusion rate of 2 *μ*g/kg/min for patients with normal organ function and at 0.5–1.2 *μ*g/kg/min for those with heart failure, liver dysfunction, and severe anasarca or patients recovering from cardiac surgery [2]. Conversely, Miyata et al. recommend that the initial infusion rate of argatroban should be reduced to 0.7 *μ*g/kg/min for Japanese patients with normal organ function and to 0.2 *μ*g/kg/min for those with liver dysfunction or having a high risk of bleeding. This has been recommended because Japanese patients treated with a dose the same as that recommended by the ACCP often experience serious bleeding accompanied by a rapid increase of aPTT [3]. When long-term anticoagulative treatment for treating HIT-associated thromboembolism is necessary, parenteral nonheparin anticoagulant treatment is usually converted to treatment with a VKA (e.g., warfarin) after platelet recovery [1, 2]. Because it takes a median of 50–85 days until HIT-related antibodies in the serum to decrease to negative levels [4], it is recommended that HIT patients without thromboembolism receive alternative anticoagulants until platelet recovery. This treatment recommendation is similar to that of HIT patients with thromboembolism. Additionally, the risk of HIT-related thrombosis is high until 2–4 weeks after the beginning of treatment; therefore, the administration of alternative anticoagulants or warfarin should be continued for at least 4 weeks [5]. Because HIT is considered to be a transient risk factor for developing VTE, at least 3 months of anticoagulant therapy is recommended in HIT patients with thrombosis. This treatment recommendation is similar to that of VTE associated with transient provoking risk factors [1, 4, 5]. Therefore, HIT patients with thrombosis require a switch to oral anticoagulants from parenteral nonheparin anticoagulants. However, an early transition to oral VKA treatment or treatment with VKA alone should be avoided because of the risk of thrombotic complications. These complications include venous limb gangrene and warfarin-induced skin necrosis, which is caused by a faster reduction of protein C than that of vitamin K-dependent clotting factors including II, VII, IX, and X [1, 6]. The 9^th^ ACCP guideline recommends that the administration of VKA be overlapped with nonheparin anticoagulant treatment for at least 5 days [1]. However, overlapping treatment with argatroban and warfarin often causes increase in the international normalized ratio and increases the risk of major bleeding [7]. We believe that two major issues associated with HIT treatment in Japan include the possibility of prolonged hospital stay and bleeding complications due to overlapping treatment with argatroban/warfarin.

Several studies have proposed the use of DOACs as new treatment options for HIT. HIT treatment with DOACs offers the following advantages: (1) DOACs have no potentially deleterious immunological interactions with HIT antibodies [8]; (2) DOACs do not reduce the levels of protein C activity, meaning that there is a lower risk of developing venous limb gangrene compared with conventional VKA therapy; and (3) DOACs have a more rapid onset compared with warfarin and could be useful for shortening hospitalization and lowering costs [2]. Warkentin et al. reported their experience with DOACs for HIT treatment in addition to a literature review. The progression or recurrence of thrombosis occurred in 2.2% (1 of 46), 0% (0 of 12), and 9.1% (1 of 11) of HIT patients treated with rivaroxaban, apixaban, and dabigatran, respectively. No major bleeding occurred in any of the patients treated with these DOACs. In these results, although efficacy and safety of rivaroxaban, apixaban, and dabigatran for HIT treatment were suggested, the data regarding the use of edoxaban for HIT treatment were not included [2].

In the Hokusai-VTE study (a randomized, double-blind clinical trial for the treatment of VTE with edoxaban), the efficacy and safety of the use of edoxaban for treating VTE were indicated, which were similar to those of other DOACs [9]. Therefore, favorable outcomes regarding the use of edoxaban to treat HIT are also expected. It is recommended that edoxaban be administered at a dose of 60 mg or 30 mg (for patients with a creatinine clearance rate of 15–50 mL/min or bodyweight below 60 kg) once per day following initial therapy with parenteral anticoagulants for at least 5 days without overlapping the treatments of the two drugs [10].

The advantages of edoxaban administration following parenteral argatroban are a lower bleeding risk, no overlap between a parenteral and an oral anticoagulant, the possibility of switching to oral anticoagulation therapy earlier, and no anticoagulant monitoring. Our patient encountered diverticulum bleeding but was treated endoscopically and had been doing well without experiencing recurrent thrombosis.

## 4. Conclusion

We successfully and safely treated a patient with HIT in the present study. To the best of our knowledge, this is the first report on the use of edoxaban for HIT treatment. The administration of edoxaban following argatroban could reduce bleeding complications and shorten the length of hospital stay. However, appropriately designed clinical studies are required to assess the effectiveness and safety of DOACs for HIT treatment.
